# Case Report: Indigenous Sovereignty in a Pandemic: Tribal Codes in the United States as Preparedness

**DOI:** 10.3389/fsoc.2021.617995

**Published:** 2021-03-15

**Authors:** Danielle Hiraldo, Kyra James, Stephanie Russo Carroll

**Affiliations:** ^1^ Native Nations Institute, University of Arizona, Tucson, AZ, United States; ^2^ College of Public Health, University of Arizona, Tucson, AZ, United States

**Keywords:** indigenous governance, COVID—19, public health, emergency preparedness, indigenous law and policy

## Abstract

Indigenous Peoples globally and in the United States have combatted and continue to face disease, genocide, and erasure, often the systemic result of settler colonial policies that seek to eradicate Indigenous communities. Many Native nations in the United States have asserted their inherent sovereign authority to protect their citizens by passing tribal public health and emergency codes to support their public health infrastructures. While the current COVID-19 pandemic affects everyone, marginalized and Indigenous communities in the United States experience disproportionate burdens of COVID-19 morbidity and mortality as well as socioeconomic and environmental impacts. In this brief research report, we examine 41 publicly available tribal public health and emergency preparedness codes to gain a better understanding of the institutional public health capacity that exists during this time. Of the codes collected, only nine mention any data sharing provisions with local, state, and federal officials while 21 reference communicable diseases. The existence of these public health institutions is not directly tied to the outcomes in the current pandemic; however, it is plausible that having such codes in place makes responding to public health crises now and in the future less reactionary and more proactive in meeting community needs. These tribal institutions advance the public health outcomes that we all want to see in our communities.

## Introduction

Combating disease, genocide, and erasure is not new to Indigenous communities in the United States and globally. Colonial efforts sought to eradicate Indigenous communities as a matter of government policy (Deloria, 1988; Coulthard, 2014; Dunbar-Ortiz, 2014). While the current pandemic affects everyone, minority and Indigenous communities in the United States experience its effects disproportionately (Hatcher et al., 2020; Hathaway, 2020; Power et al., 2020; Rodriguez-Lonebear et al., 2020). To combat these effects, Native nations have asserted their inherent sovereign authority to protect their citizens (Curley, 2020; Gunderson, 2020; Lakhani, 2020; Walker, 2020). Whether these assertions are made by declaring states of emergency, securing borders, or adopting public health codes, Native nations are demonstrating the importance of having public health and emergency preparedness infrastructures in place to effectively meet community needs during this global public health crisis.

While a few nations have drafted and adopted public health and emergency codes during the pandemic, some Native nations have been building or using existing infrastructure to address the 2019 novel coronavirus (COVID-19) crisis. For example, Lummi Nation implemented a rapid response using its existing Emergency Health Powers Code adopted in 2017. The Lummi Nation began preparing a COVID response in February 2020 when the vast spread of the virus appeared inevitable. Lummi elected leaders and health officials began gathering medical supplies including test kits and arranging for test processing (Mapes 2020). COVID-19 on the Navajo Nation has attracted national media and highlights nation-specific efforts to address the public health emergency, which included creating communication materials in Navajo and declaring a public health emergency order in mid-March that prohibited mass gatherings and advised citizens to stay at home (Nation, 2020). Six months later, Dr Anthony Fauci, director of the National Institute of Allergy and Infectious Diseases, has publicly stated that the Navajo efforts could serve as a response model (Armas, 2020). These stories and the many others demonstrate the importance of tribal public health systems during a national crisis to better position Native nations to assert their sovereignty in the best interests of their citizens and communities.

### Tribal Public Health and Framework for Tribal Public Health Law

Scholars are pushing back on the deficit narrative that focuses on disparities, inequities, and disadvantages in tribal public health (Tingey et al., 2016; McKinley et al., 2019). Hoss (2019a) begins to shift this narrative by providing a framework for tribal public health law that includes four pillars: 1) Native nations are inherently sovereign; 2) federal Indian law impacts intergovernmental relationships among Native nations, states, and the federal government (and, as a result, public health); 3) Native nations exert authority through tribal law; and 4) interventions without tribal consent can further undermine public health and promote structural violence. We situate this brief research report in this framework to understand the public health institutional capacity that exists in Indian Country during this current pandemic. We add to this discussion the importance of incorporating Indigenous Data Sovereignty and the role of the CARE Principles for Indigenous Data Governance for outside researchers, governments, and organizations when Native nations are adopting public health and emergency preparedness policies (RDA International IDSov IG, 2019).

Indigenous Data Sovereignty express a nation’s authority and jurisdiction over information derived from its territories, citizens, communities, and interests (Kukutai and Taylor, 2016; Snipp, 2016; Rainie et al., 2017). Indigenous data governance activates Indigenous data sovereignty by aligning the collection, application, use, and stewardship of Indigenous Peoples’ data with their values, cultures, and interests (Walter et al., 2018; Carroll et al., 2019). The rapid response that occurs during times of emergencies such as the COVID-19 pandemic does not excuse outsiders from disregarding or neglecting to follow Indigenous data sovereignty practices. During this time, it is even more important to practice good data governance and build partnerships that honor tribal sovereignty. Such practices and partnerships fall within the CARE Principles framework, which affirms Collective Benefit, Authority to control, Responsibility, and Ethics (RDA International IDSov IG, 2019). The UN Special Rapporteur on the rights of Indigenous Peoples, José Francisco Cali Tzay (2020), emphasizes engaging representative institutions during this time; however, this warning assumes infrastructure and capacity exists in most communities. As we begin to examine existing public health and emergency infrastructure, we see there are areas for Native nations to grow and develop to meet the demand (Groom et al., 2009).

Governance of tribal public health sits outside of federal and state authority; however, in no way does this recognition relieve the United States from its treaty obligations and trust responsibilities to Indigenous Peoples. In the 1980s, as control over health care services and facilities began to shift from the federal government to the tribes, the predominant focus on treatment began to change. The Indian Health Service (2015) (IHS), an agency within the Department of Health and Human Services responsible for providing federal health services to federally recognized tribes, began to invest in preventative care and environmental services (Sequist et al., 2011). This period saw a growing number of tribes governing health services according to their own needs and assuming a larger role in the delivery of health care to their own peoples. This trend was concurrent with increased attention to preventative programs as well as to behavioral and mental health issues (Rainie et al., 2015).

Since the Indian Self-Determination and Education Assistance Act of 1975 (ISDEAA, P.L. 93–638) and its amendments, the United States government has met its obligations to provide health care through direct services or the provision of funds for tribes or other American Indian organizations to provide services. The ISDEAA and amendments began a shift in tribal control of IHS health care funding and facilities (Rainie et al., 2015). A series of amendments to the ISDEAA—P.L. 100-472 in 1988, the Indian Health Care Amendments of 1992 (P.L. 102–573), and the Tribal Self-Governance Amendments of 2000 (P.L. 106-260)—enhanced tribal control through the creation of self-governance compacts for health care services that provide money through block grant-like mechanisms for Native nations to administer programs and design services to meet tribal priorities (Dixon, 2001; Rainie et al., 2015). In essence, the amendments gave tribes the right to decide how to use federal funds. IHS administers the contracting and compacting processes provided under the ISDEAA and Tribal Self-Governance Act. As of 2016, IHS had negotiated 90 self-governance compacts with federally recognized Tribes.^1^ In 2017, through P.L. 93-638 Self-Determination contracts, Native nations and Alaska Native corporations administered 19 hospitals, 284 health centers, 79 health stations, and 163 Alaska village clinics.^2^ This shift in federal policy has broadened public health and emergency opportunities for Native nations to proactively meet the health needs of their communities rather than constantly having to react to the emergency at hand.

The federal government acknowledges that the 574 federally recognized Native nations maintain sovereign authority to build and sustain public health systems that are part of the broader patchwork of public health authority across the United States (see 45 C.F.R. 164.501). While tribal control of health care services and facilities has increased, a need remains for investments in tribal public health infrastructure in order to monitor public health, address emerging needs, provide services, and create informed policies (Warne, 2005; Tribal Epidemiology Centers, 2013). In addition, Native nations see challenges to exercising that authority such as building public health infrastructure; establishing relationships, roles, and responsibilities with state, county, and local health departments; and data and information sharing (Richmond and Ross, 2009; Hernandez and Robinson, 2014). To do this, nations need access to resources. Oftentimes this means access to federal programs and services that are vastly underfunded. In the United States., health inequities between Indigenous and non-Indigenous mainstream populations have existed for decades (Rhoades et al., 1987; Brenneman et al., 2000; Johnson and Rhoades, 2000). As a result, we see variation in the effectiveness of tribal public health systems in Indigenous communities in the United States.

During a crisis, emergency infrastructure constitutes an important companion to public health systems. The direct relationship between Native nations and the United States Federal Emergency Management Agency (FEMA) is fairly new. The Robert T. Stafford Disaster Relief and Emergency Assistance Act (Stafford Act) of 1988 authorizes the United States’ President to declare an emergency or major disaster in situations where the responses are beyond the capacity of a state or local government. Since the Sandy Recovery Improvement Act of 2013 (P.L. 113-2), “The Chief Executive [or Governor] of an affected Indian tribal government may submit a request for a declaration by the President that a major disaster exists consistent with the requirements.” Prior to this law, tribal governments had to rely on state governors to request a presidential declaration on their behalf (Sunshine and Hoss, 2017). However, Native nations were not simply waiting on the United States to intervene when addressing emergency or disaster efforts in their communities. Native nations have passed emergency management plans as early as 1990 in the case of the Navajo Nation (2020). Since 2013, more than 40 Native nations have directly requested presidential declarations to address areas of COVID-related matters, severe storms and flooding, and other natural disasters.^3^ Sunshine and Hoss (2017) argue that Native nations might consider using emergency declarations as “critical” public health tools to access resources and loosen the bureaucratic red tape that often impedes a nation’s ability to respond. By passing emergency tribal laws, Native nations assert another level of governing authority to respond to public health crises.

Native nations continue to address the neglect and failure of the federal government in living up to its treaty and trust responsibilities. In doing so, Native nations are developing sophisticated healthcare, public health, and emergency systems that are grounded in their own understanding of how to address community needs and in their own values and beliefs. Examining tribal public health and emergency codes provides a lens to examine Native nation innovation for self-determination and governance during a public health crisis.

### Tribal Public Health Codes

More policy research is needed on tribal public health and emergency management. The enactment of tribal public health and emergency codes has occurred at a lower rate than the codification of other governmental authorities such as economic development and law and order. In 2014, the National Congress of American Indians (NCAI) found 520 public health-related codes that fell into 10 different categories: agriculture and food safety; alcohol, tobacco and other drugs; animal management and control; emergency planning and management; environmental health; health data; health services; health systems governance; infectious disease management; injury and violence prevention; public health infrastructure; and health and cultural resource protections. As NCAI notes, many public health provisions adopted by Native nations are often framed around public safety. Bryan et al. (2009) conducted a similar study finding 56 publicly available tribal codes that address public health. Similar to NCAI, the authors categorized the codes into broad themes: “environmental health and sanitation; public safety and injury prevention; protection from violence and abuse; substance abuse, mental illness, and tobacco; communicable disease control, surveillance, and research; and other.” These studies begin to provide more evidence of the ways in which Native nations are actively asserting their sovereign rights around public health and emergency management in preparedness to meet these needs. Additionally, in the mainstream United States, the Centers for disease Control and Prevention’s Tribal Emergency Preparedness Law (2017) provides an understanding of what is needed during emergencies such as incident command centers, points of contact, and health communication systems.

During the COVID-19 pandemic, many public virtual convenings have featured tribal leaders sharing their nation’s response to this virus such as National Congress of American Indian’s (NCAI) Tribal Governments in Action webinar series.^4^ Some of the tribal leaders and employees have mentioned that having a tribal law in place helped coordinate the nation’s response efforts, which led us to survey the types of public health and emergency laws that exist and the areas in which Native nations are asserting their authority and preparing the resilience of their communities to combat the destruction attending this disease.^5^ Given that public health and emergency preparedness are connected in a public health crisis, we argue that natural/climate, public health, and safety preparedness should all be linked in the institutions that nations are building rather than siloed.

## Methods

In this pilot study, during the months of May–September 2020 we collected and thematically coded 41 tribal public health and emergency preparedness codes from 37 federally recognized Native nations in the United States to gain a better understanding of the institutional public health and emergency capacity that exists during COVID-19. We searched and accessed any publicly available codes through the Tribal Law Gateway on the National Indian Law Library and the Tribal Court Clearinghouse databases. To make sure that we exhausted all databases, we conducted a google search using “tribal public health code” and “tribal emergency code” key terms. The code titles range from Communicable disease, Vaccination, and Quarantine Ordinance, Community Health, Emergency Management and Homeland Security to Environmental and Public Health Ordinance, Health and Safety, Health and Sanitation. Using an iterative process geared toward pandemic public health response and based off previous work by Hoss (2019b), CDC (2017), NCAI (2014), and Bryan et al. (2009), we identified twelve themes: environment, crimes against health, health communications, quarantine and isolation, incident command systems, point of contact for tribal public health issues, sovereignty/governance, culture, emergency, communicable diseases, self-governance compact, and data sharing. Some of the themes represent common trends that we observed after reviewing the codes such as crimes against health (separate from a nation’s criminal code), communicable diseases, and environment.

Using a dichotomous 0/1 coding, two researchers read each code and coded the eleven themes using 0 to represent not addressing the theme and 1 as affirming the theme. Any differences were resolved in conversation or by a third researcher’s input. To understand when most of the activity occurred, we documented the year when the last activity occurred, being either amendment or enactment. We understand that Native nations are not required to post their own nation’s law for public consumption and there may be many instances where some nations have enacted public health and emergency laws and chosen not to share them publicly. Therefore, those tribal public health and emergency codes that are not made publicly available by Native nations are not included in this dataset.

We used a narrow search and coding process. As a result, we did not include articles or chapters that were not specific to public health and/or emergency preparedness. For instance, there are multiple health related mentions in tribal law and order codes, other criminal offenses codes, agriculture and food and safety, and sanitation concerns--as can be found in the NCAI (2014) data--that are not included in this dataset. Some outcomes of interest are the number of codes specific to Indigenous data sovereignty, culture, and geographic location of Native nations enacting codes. Our sole focus for this collection was to identify standalone tribal public health codes in an attempt to assess institutional capacity within Indian Country for public health disease surveillance, protection, and emergency preparedness, and less around criminalization within public health (Hoss, 2019b). We parsed the codes into two categories those enacted before 2020 and those enacted during the 2020 timeframe in which we conducted the search.

## Results

Out of the 41 codes collected, only seven (17%) Native nation codes are specific to emergency preparedness and management in all years; however, 17 (47.1%) codes (public health and emergency) have emergency provisions included (Table 1). More than half (*n* = 23, 56.1%) of the Native nations have pre-existing self-governance compacts, which suggests that as nations are acting on a nation-to-nation basis with the federal government, they are building institutions to support their sovereign efforts to address the welfare of their citizens. Of the codes enacted prior, from 1988 to 2019, more are likely to address environment (*n* = 18, 54.5%). Of the 8 (100%) codes enacted in 2020, all addressed communicable diseases and quarantine and isolation. More than half of the codes passed in 2020 included provisions for emergencies (*n* = 5, 62.5%), health communications (*n* = 6, 75%), and point of contact (*n* = 6, 75%). In addition, half of the nations that passed codes in 2020 have self-governance compacts (*n* = 4, 50%) with IHS. Of the nine codes that included data sharing provisions for all years, five of those codes were passed in 2020.

**TABLE 1:** Frequency of public health and emergency preparedness themes in tribal public health codes 1988-2020 (*N* = 41).

Frequency table	1988-2019 (33)		2020 (8)		Total all years (41)	
Themes	n	%	n	%	n	%
Communicable diseases	13	39.4%	8	100.0%	21	51.2%
Crimes against health	9	27.3%	1	12.5%	10	24.4%
Culture	4	12.1%	1	12.5%	5	12.2%
Data sharing	4	12.1%	5	62.5%	9	22.0%
Emergency preparedness	12	36.4%	5	62.5%	17	41.5%
Environment	18	54.5%	0	0.0%	18	43.9%
Health communications	9	27.3%	6	75.0%	15	36.6%
Incident command systems	2	6.1%	3	37.5%	5	12.2%
Point of contact for tribal public health issues	13	39.4%	6	75.0%	19	46.3%
Quarantine and isolation	7	21.2%	8	100.0%	15	36.6%
Self-governance compacts	19	57.6%	4	50.0%	23	56.1%
Sovereignty/Governance	12	36.4%	3	37.5%	15	36.6%

To understand outbreak response and public health capacity during times of pandemic and emergencies, we created a subset of analysis that examines the emergency provisions (*n* = 17, 41.5%) found in the dataset, eight (47.1%) included a response to outbreaks and five (29.4%) respiratory surveillance (Table 2). We see Native nations including specific public health capacity arrangements that complement emergency preparedness. For example, of the nine (22%) data sharing points found in the dataset, eight (47.1%) of those are included in codes that also address emergency preparedness. The same is true for health communications and point of contact provisions. Of the fifteen (36.6%) total health communications points found, ten (58.8%) accompany emergency provisions. For tribal public health point of contact, twelve (70.6%) of the nineteen (46.3%) are included in public health codes that also address emergencies.

**TABLE 2:** Frequency of emergency preparedness themes in tribal public health codes 1988-2020 (*N* = 17).

Subset of emergency preparedness (n = 17)		
Themes	n	%
Data sharing	8	47.1%
Health communications	10	58.8%
Outbreak response	8	47.1%
Point of contact for tribal public health issues	12	70.6%
Respiratory surveillance	5	29.4%

**FIGURE 1:**
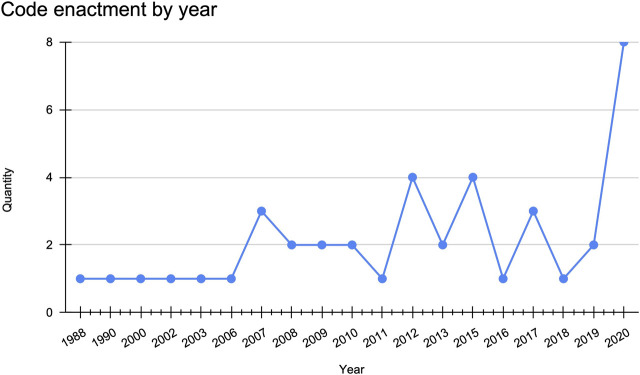
Tribal public health code enactment by year (*N* = 41).

From 2006 to 2013, there was a steady stream (*n* = 17, 41.4%) of code enactment or amendments (Figure 1). As expected, we see a peak in activity in 2020 (*n* = 8 out of 41, 19.5%) where the largest number of codes were either enacted or amended in a single year.

**FIGURE 2:**
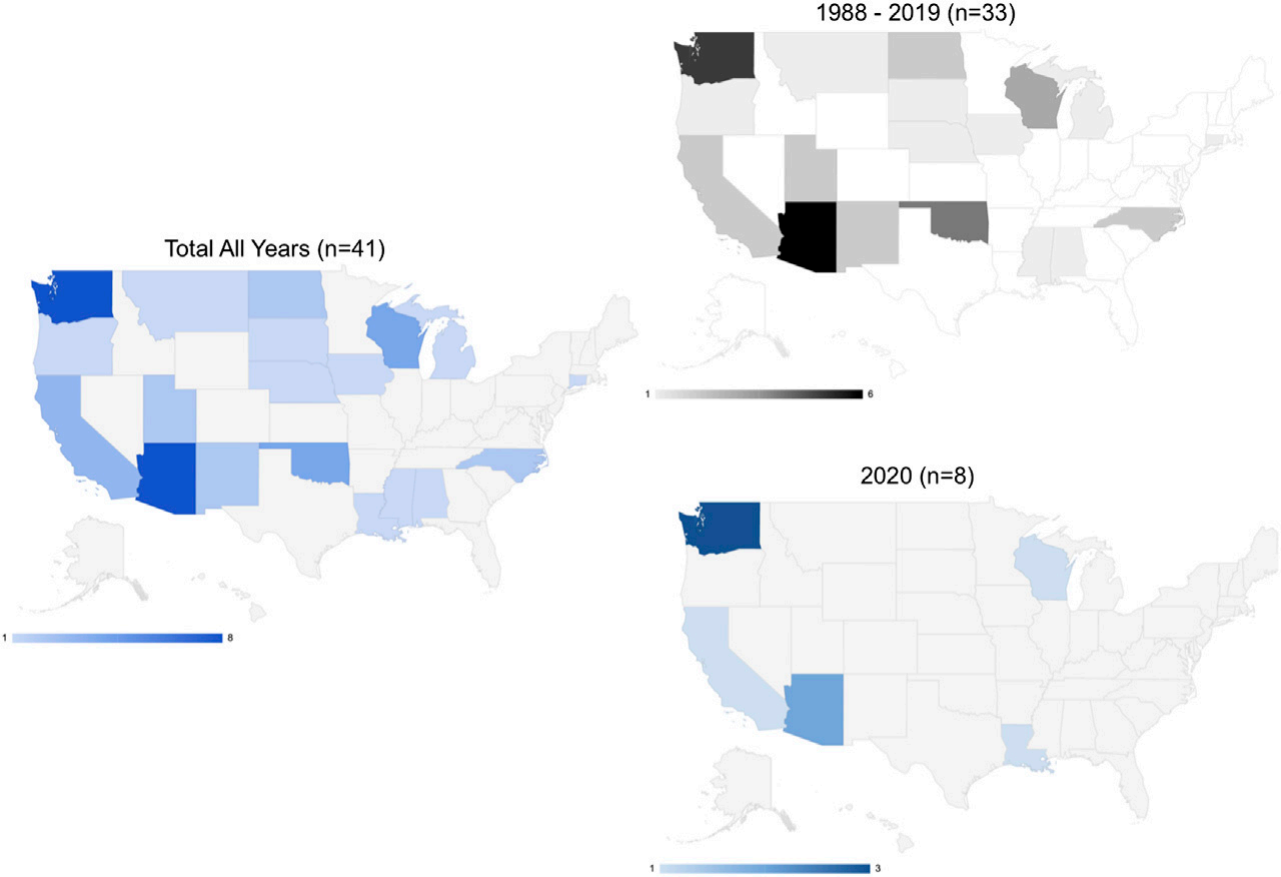
Tribal public health codes by state (*N* = 41).

Most of the activity for code enactment or amendment has occurred in the West and Midwest census regions with Native nations sharing geography with the state of Washington having the most activity (Figure 2). In the West, we see the greatest mix of types of enacted codes being both public health and emergency-specific.

In 2020, we see code enactment occurring in some of the harder hit states (Johns Hopkins University and Medicine, 2020) such as Arizona, California, Louisiana, and Washington (Figure 2).

### Discussion: Lessons Learned From Tribal Codes

Surveying publicly availably codes provides insight into ways Native nations might approach public policy. The examples below offer broad considerations--that are by no means are exhaustive--for Native nations interested in strengthening tribal public health and emergency management governance.

### Separate Public Health and Emergency Management Codes

Of the 37 Native nations, four Native nations have enacted separate public health and emergency management codes. One nation passed a public health code and a separate communicable disease code while another passed a public health quarantine and isolation code. Seven Native nations opted to pass Health and Safety codes, which typically cover animal control, fireworks, public safety, and sanitation issues. To further back up the nation’s public health code, the Pascua Yaqui Tribe (PYT) adopted a Public Health Emergency Preparedness Plan that includes information on its health care delivery system, legal authorities, and contractual partners among other points. PYT explains in its plan that it is a living document that is subject to annual review and revisions.

### Communicable Diseases

Considering the current pandemic, we sought to identify how many of the 41 codes specifically addressed communicable diseases (Table 1); 51% (*n* = 21) of the codes mention communicable diseases. In 2007, in addition to its Public Health and Safety code, Stockbridge-Munsee adopted a Communicable disease, Vaccination, and Quarantine Ordinance that states the need “to prepare for possible bioterrorism issues and other communicable disease issues” and “if this area is left unaddressed, the political integrity, economic security, and the general health and welfare is threatened” (Stockbridge-Munsee Mohican Tribe). Jamestown S’Klallam Tribe’s Public Health and Safety code states, “Tribal health care providers will assist local health officials in identifying exposed contacts of a communicable disease, when necessary, and assure appropriate testing, treatment, or chemoprophylasis [sic] is carried out” (Jamestown S’Klallam Tribe, 2020). The Chitimacha Tribe of Louisiana includes a tracking section which states, “The Public Health Authority shall ascertain the existence of an illness or health condition that may be a potential cause of a public health emergency, investigate all such cases for sources of infection and to ensure that they are subject to proper control measures, and define the distribution of the illness or health condition” (Chitimacha Tribe of Louisiana, 2021). The Chitimacha code underscores what many public health officials have advocated for during the current pandemic. Not only is it important to report cases but it is important to track and trace to better contain a virus.

### Public Health and Emergency Communications

In other areas of emergency, we see some nations including health communication systems and even fewer providing provisions for incident command systems. San Manuel Band of Mission Indians’ Emergency Management Ordinance adopts the National Incident Management System using an incident command system to manage all emergencies within the nation’s reservation. The ordinance states that once the Business Committee, the Chairperson, or its successor determines that a state of an emergency exists, they have the authority to declare an emergency (San Manuel Band of Mission Indians, 2020). Codifying how a nation organizes in times of emergencies allows the government to respond according to plan rather than react to meet the crisis at hand.

### Sovereignty Authority and Culturally Appropriate Language

While the act of passing a tribally specific code is an assertion of tribal sovereignty, we were interested in seeing how many tribal codes explicitly mentioned the inherent sovereign authority of the nation’s government. Fifteen codes explicitly affirm a nation’s authority. Other affirmations might be interpreted by the existence of self-governance compacts. Twenty-three (56.1%) of the Native nations included in this dataset have negotiated self-governance compacts with the federal government, which demonstrates a nation backing up its sovereign authority to negotiate a compact by passing its own rules and laws.

On the other hand, only five codes mention the distinct culture or belief system that guides the nation and serves as a foundation for sovereignty. The Confederated Tribes of the Umatilla Indian Reservation (CTUIR) includes a Tribal Health Philosophy in its Environmental Health and Safety Code. The section reads, “Spiritually, we do not separate ourselves from the surrounding natural world. Therefore, the land, air, water and natural resources of the Umatilla Reservation must be maintained in a healthy and safe condition to sustain all forms of life using both traditional ways and modern technology. We recognize that the responsibility to intervene in human activities that create an unhealthy imbalance in nature is essential to protecting all natural resources” (Confederated Tribes of the Umatilla Indian Reservation, 2020). Here, CTUIR is articulating to its citizens and outsiders that the health decisions that are made align with its collective responsibility to the natural world.

### Indigenous Data Sovereignty and Data Governance

In a time where access to health data is increasingly a challenge, we would expect to see more mentions of health data governance in the more recently passed public health and emergency codes. However, that is not the case. Of the 41 codes adopted, only nine (22%) include data sharing provisions. With respect to reporting authorities, the Yurok Tribe’s Public Health Ordinance states, “The Yurok Public Health Officer is authorized to report to a local health department, State Department of Public Health, and/or the Indian Health Service any information concerning a reportable disease or condition, an unusual cluster, or a suspicious event that they reasonably believe has the potential to be caused by or an indicator or bioterrorism” (Yurok Tribe’s Public Health Ordinance, 2020). Yurok public health officials have the authority to report public health concerns to outside governments; however, the code does not mention power to enter health data agreements. In March 2020, the National Indian Health Board (NIHB) surveyed the ability of tribal leaders, providers, and partners to adequately address the COVID-19 emergency. In addition to federal and state communication, and diagnostics testing, respondents listed one of the anticipated challenges as “planning for outbreak management” (National Indian Health Board, 2020). Indigenous Data Sovereignty affirms Native nations rights to outside and nation-specific data, and pandemic and pre-pandemic guidance asserts these nations’ need to access data in order to effectively plan for and manage potential outbreaks (Carroll et al., 2019; Rainie et al., 2017; Urban Indian Health Institute, 2020).

### Preparedness During Times of Crisis

The code enactments and amendments from 2006 to 2013 coincide with the H1N1 pandemic that occurred in 2009 and ended in late 2010 (Figure 1). However, from 2006 to 2009 timeframe, the Sac and Fox Tribe of the Mississippi in iowa (Health and Safety code passed in 2007) is the only nation in the dataset to have included provisions for disease outbreaks in its code, which are specific to notification procedures. Snoqualmie Tribe’s Emergency Management Department code defines its scope as “to mitigate, prepare for, respond to, and recover from injury and damage to persons or property resulting from emergencies or disasters, whether natural or man-made” (Tribe Snoqualmie, 2021). Whereas, as mentioned previously, Stockbridge-Munsee Tribal Law, 2007 Communicable disease, Vaccination, and Quarantine Ordinance states the need to prepare for bioterrorism. We can infer from the data that as Native nations are incorporating emergency provisions in their codes, tribal officials are making the effort to address the capacity in which these emergencies might be met. Among the codes adopted or amended in 2020, we see Native nations creating institutions to sustain them during the pandemic particularly in two states where governors were arguably more resistant to act according to CDC recommendations (Figure 2).

## Conclusion

Generally, we find that many of the codes use mainstream health and safety, and emergency language. In some instances, Native nations are adopting language from the state in which they share geography or explicitly adopting state law in these areas of law making. There is great opportunity for Native nations to position these codes to align with their own cultural values and understanding of how to address community challenges. Native nations are addressing governance challenges in other areas that can be transferred or applied in public health and emergency settings (Hiraldo et al., 2020).

Native nations are building culturally appropriate public health and emergency institutions to reinforce inherent sovereign rights and establish a standard of community public health. While the existence of institutions is not directly tied to the outcomes found during the current pandemic, it is possible to argue that having such codes in place make responding to public health and emergency crizes now and in the future less reactionary and more proactive in meeting community needs. As the enactment of tribal public health codes evolves, Native nations are acting responsively to emerging needs. We view these institutions as advancing the public health outcomes that we all want to see in our communities.

## Data Availability

The original contributions presented in the study are included in the article further inquiries can be directed to the corresponding author.

## Footnotes

1 Tribal Self-Governance. Indian Health Services, https://www.ihs.gov/sites/newsroom/themes/responsive2017/display_objects/documents/factsheets/TribalSelfGovernance.pdf. Accessed September 25, 2020.

2 Quick Look. Indian Health Services, https://www.ihs.gov/newsroom/factsheets/quicklook/. Accessed September 25, 2020.

3 Tribal Affairs. Federal Emergency Management Agency, https://www.fema.gov/about/organization/tribes. Accessed September 26, 2020.

4 National Congress of American Indians. COVID-19 Resources for Indian Country. COVID-19 Forums and Webinars, http://www.ncai.org/Covid-19/resources-for-indian-country/ncai-covid19-webinars [Accessed September 29, 2020].

5 Seven Directions. Gathering Grounds Community Meeting IV: A Look at Lummi Nation's Response to COVID-19, https://www.indigenousphi.org/covid-resources/a-look-at-lummi-nations-response-to-covid-19 [Accessed September 29, 2020].
